# The Development of Al^18^F-NOTA-FAP-2286 as an FAP-Targeted PET Tracer and the Translational Application in the Diagnosis of Acquired Drug Resistance in Progressive Prostate Cancer

**DOI:** 10.3390/pharmaceutics17050552

**Published:** 2025-04-23

**Authors:** Xia Du, Yu Zhang, Yao Jia, Bo Gao

**Affiliations:** 1 Department of Radiology, The Affiliated Hospital of Guizhou Medical University, Guiyang 550001, China; duxia05@126.com (X.D.); ayyao01233@gmail.com (Y.J.); 2 Department of Nuclear Medicine, Shengli Clinical Medical College of Fujian Medical University, Fujian Provincial Hospital, Fuzhou University Affiliated Provincial Hospital, Fuzhou 350001, China; zhangyu1993@fzu.edu.cn; 3 Key Laboratory of Brain Imaging, Guizhou Medical University, Guiyang 550001, China

**Keywords:** fibroblast activation protein-α, pre-chelation, tumor heterogeneity, Al^18^F-NOTA-FAP-2286, prostate cancer, acquired resistance

## Abstract

**Objectives**: Tumor heterogeneity and acquired resistance to prostate-specific membrane antigen (PSMA) radioligand therapy (PRLT) pose significant challenges to PSMA PET-based diagnosis. This study aimed to develop an Al^18^F-labeled FAP-targeted tracer and explore the diagnostic value in acquired drug-resistant tumor models. **Methods**: To identify potential targets for imaging drug-resistant prostate cancer, bioinformatic analysis was employed to correlate FAP expression levels with genes associated with tumor progression and radiotherapy resistance. Molecular docking technology simulations were utilized to screen FAP ligands for optimal binding affinity and target specificity. The most promising ligand, FAP-2286, was radiolabeled with ^18^F to develop a novel PET imaging agent, Al^18^F-NOTA-FAP-2286 PET. To evaluate the diagnostic potential of this agent, various tumor models were established. U87 cells were used to optimize the imaging protocol and assess targeting efficiency and 22RV-1-resistant cells co-xenografted with NIH-3T3 cells were used to model acquired drug-resistant prostate cancer. The diagnostic efficacy of Al^18^F-NOTA-FAP-2286 PET in this acquired drug-resistant model was assessed and validated through immunohistochemical staining of tumor tissue. **Results**: Bioinformatic analysis confirmed the association between FAP expression and key genes involved in radiotherapy resistance, such as *HIF1α*, *BCL2*, *ATM*, and *EGFR*. Molecular docking studies demonstrated the strong binding affinity of FAP-2286 to FAPα (−10 kcal/mol). Al^18^F-NOTA-FAP-2286 PET/CT imaging in U87 tumor-bearing mice revealed accurate targeting of high FAP-expressing xenografts. The imaging characteristics of Al^18^F-NOTA-FAP-2286 were comparable to ^18^F-FDG and ^68^Ga-FAP-2286 but with a prolonged imaging window compared to ^68^Ga-FAP-2286. In acquired drug-resistant prostate cancer xenograft nude mice, Al^18^F-NOTA-FAP-2286 could effectively detect tumor lesions, as confirmed by immunohistochemical analysis. **Conclusions**: Al^18^F-NOTA-FAP-2286, as a PSMA-independent imaging agent, holds promise as a valuable complementary molecular imaging tool for assessing acquired resistance to PRLT.

## 1. Introduction

The clinical course of prostate cancer (PCa) is highly heterogeneous, ranging from an indolent to an aggressive phenotype [1]. Interpatient heterogeneity manifests as varying rates of disease progression, while intratumoral heterogeneity encompasses a diverse array of molecular subtypes. For instance, some patients exhibit a slow, indolent disease course, whereas others rapidly progress to fatal metastatic disease. PCa can harbor multiple molecular subtypes, including TMPRSS2-ERG fusions, SPOP mutations, and others [2]. The prolonged natural history of PCa, coupled with its inherent heterogeneity, poses significant challenges in accurate diagnosis, staging, and assessment of treatment response [3]. To address these challenges and guide optimal therapeutic decisions, precise diagnostic strategies are essential to manage the heterogeneous presentation of PCa [4]. Molecular imaging techniques, particularly nuclear medicine imaging targeting specific bioactive markers, offer the potential to capture diverse aspects of the tumor phenotype, providing enhanced and personalized information [5].

Prostate-specific membrane antigens (PSMAs) are frequently overexpressed in PCa [6], enabling non-invasive in vivo monitoring of PSMA expression and PCa malignancy through modalities, such as PSMA-targeted positron emission tomography (PET). Currently, PSMA PET is primarily employed to detect recurrent PCa, with most clinical studies validating this application. Compared to traditional technetium-99m-methyl-diphosphonate (^99m^Tc-MDP) bone scanning, PSMA PET offers earlier detection of metastatic PCa in patients post-radical prostatectomy, influencing treatment decisions in approximately half of cases [7,8,9]. However, inter- and intra-lesional heterogeneity, as well as the emergence of PSMA-negative tumor characteristics, can lead to false-negative imaging results [10]. Similarly, the development of resistance to PSMA radioligand therapy (PRLT) can complicate the interpretation of PSMA PET [11]. Previous studies have demonstrated that PSMA PET may yield false-negative results when PSMA-negative tumor areas exceed 50% on immunohistochemistry (IHC) despite high prostate-specific antigen (PSA) expression [12]. In addition, up to 15% of bone metastases may exhibit PSMA-negative characteristics [13]. False-negative PSMA PET results can compromise staging accuracy and efficacy assessment in PCa patients. Given the inherent heterogeneity of PCa and the risk of false-negative results with unimodal imaging, there is a pressing need for personalized diagnostic strategies and the development of novel in vivo diagnostic protocols targeting heterogeneous or acquired resistance characteristics.

As previously mentioned, acquired drug resistance complicates tumor characterization. While multi-omics imaging, combining PSMA, glucose metabolism, and tumor microenvironmental markers, could potentially enhance the characterization of PCa heterogeneity, its practical implementation in a single imaging examination remains challenging. Although ^18^F-FDG PET/CT is widely employed as a core technology in nuclear medicine imaging to aid in clinical staging, efficacy assessment, and prognosis of tumors, its role in PCa imaging is limited due to the typically low glucose metabolism of prostate tumors [3]. However, alterations in glucose metabolism during PCa progression, such as the upregulation of GLUT transporter proteins, have informed the use of ^18^F-FDG PET in advanced disease [14]. In addition, several studies have shown that ^18^F-FDG PET can detect lesions that are negative on ^68^Ga-PSMA PET in certain PCa cases [15,16]. While the underlying mechanism for this discrepancy remains unclear, it may involve neuroendocrine differentiation of tumor tissue or other factors that induce increased glycolysis. Although ^18^F-FDG is not optimal for routine PCa imaging, these findings suggest that it may serve as a complementary modality in patients with biochemical evidence of disease but negative ^68^Ga-PSMA PET scans. To some extent, ^18^F-FDG PET may be a valuable complementary imaging tool for PCa patients with low or no PSMA expression.

For optimal evaluation and treatment of malignant tumors, a promising strategy involves imaging and intranuclear radiation therapy targeting fibroblast activation protein-α (FAP). Imaging with FAP inhibitor (FAPI) analogs may aid in characterizing the tumor microenvironment [17]. Recently, quinoline-based FAPI compounds have demonstrated promising results in diagnosing various cancer types, including PCa, making them a focal point of research in nuclear medicine and molecular imaging. While the role of FAP in PCa remains relatively unexplored, it appears to possess the potential to identify PSMA-negative or low-expressing lesions [18]. Clinically, PSMA-negative tumors and acquired drug-resistant tumors pose challenges to PSMA PET-based diagnosis, necessitating the development of strategies to overcome these limitations and establish a more precise pathological profile for personalized treatment. More importantly, glucose metabolism and tumor mesenchymal fibrosis are pivotal factors in the progressive development of tumor cells and the tumor stroma. In this study, we used ^18^F-labeled FAP as a complementary imaging agent and validated its diagnostic utility in an acquired drug-resistant tumor model, offering a novel perspective for personalized PCa treatment.

## 2. Materials and Methods

### 2.1. Materials and Reagents

^68^Ga-FAP-2286 and ^177^Lu-PSMA-617 were synthesized following standard protocols [19,20], with radiochemical purity (RCP) strictly maintained above 95%. ^18^F-FDG was procured from Shanghai Atom Kexing Pharmaceutical Co., Ltd. (Shanghai, China). U87, 22RV-1, and NIH-3T3 cell lines were obtained from the Cell Bank, Chinese Academy of Sciences (Shanghai, China). RPMI 1640 and DMEM cell culture media were purchased from Thermo Fisher Scientific Co., Ltd. (Rockford, IL, USA). All other necessary materials, solvents, and chemicals were purchased from Shanghai Titan Technology Co., Ltd. (Shanghai, China).

### 2.2. Bioinformation Analysis

#### 2.2.1. Data Sources of Bioinformatics Analysis

RNA sequencing (RNA-Seq) data and associated clinical information for prostate adenocarcinoma (PRAD) were acquired from the Cancer Genome Atlas (TCGA) program database (https://portal.gdc.cancer.gov/ (accessed on 16 September 2024)). Clinical data within the TCGA-PRAD dataset, including sample type, lymph node metastasis status, and Gleason score, were extracted utilizing the “rjson” package. FAP gene expression levels were assessed across three cohorts: (1) normal tissues (n = 52) and primary tumor tissues (n = 497); (2) normal tissues (n = 52), patients without lymph node metastasis (N0, n = 345), and patients with lymph node metastasis (N1, n = 79); and (3) normal tissues (n = 52) and patients with Gleason scores of 6 (n = 45), 7 (n = 247), 8 (n = 64), 9 (n = 136), and 10 (n = 4). Gene expression levels of FAP were quantified in each cohort using transcripts per million (TPM) as the unit of measurement.

FAP expression across different groups was visualized using box plots generated with the “ggplot2” package in R. These plots displayed the median, interquartile range, and outliers for each group, facilitating a comparison of FAP expression distributions. The analysis explored potential relationships between FAP expression and clinicopathological parameters, including PCa sample type, lymph node metastatic status, and disease progression as assessed by Gleason score.

#### 2.2.2. Correlation Analysis Based on Genes Associated with Radiotherapy Resistance

Correlation analysis was conducted to investigate the relationship between FAP and genes associated with radiotherapy resistance. A curated list of radiotherapy resistance genes was compiled from the relevant literature and established databases. Hierarchical clustering order (HCO) correlation analysis of FAP and these resistance genes was then performed using the “corrplot” package within the R statistical programming language. The resulting correlation coefficients are visually represented using a color gradient, ranging from blue to indicate negative correlations to red for positive correlations.

To investigate the relationship between FAP expression levels and the expression of radiotherapy resistance-related genes, samples were dichotomized into high and low FAP expression groups based on the median FAP expression value. Differences in radiotherapy resistance gene expression between these groups were then assessed using the Wilcoxon rank-sum test, implemented via the ggboxplot function within the ggpubr R package. The statistical significance levels were set at *p* < 0.05, *p* < 0.01, and *p* < 0.001, respectively.

In addition, the correlation between FAP expression and the expression levels of key radiotherapy resistance-related genes, including hypoxia-inducible factor 1α (HIF1α), B-cell lymphoma 2 (BCL2), ATM serine/threonine kinase (ATM), and epidermal growth factor receptor (EGFR), was analyzed using Pearson’s correlation coefficient. The “ggscatter” function was employed for visualization, and the statistical significance of each correlation was determined by calculating the corresponding *p*-value.

### 2.3. Molecular Docking of FAP-Targeting Small Molecule Imaging Agents

A total of six major FAP-targeted imaging agents were selected as potential research objects for molecular docking simulations. Molecular docking techniques were employed to elucidate the interactions between FAP ligands and FAP alpha proteins. The three-dimensional structure of FAP alpha was obtained from Protein Data Bank (PDB) file 1Z68, downloaded from the RCSB PDB, cleaned, and prepared for docking. FAP ligands, serving as ligands, underwent structural definition, and molecular docking simulations were performed using AutoDock Vina [21] employing the Lamarckian genetic algorithm (LGA) within a specified search space encompassing the putative FAP alpha binding site. The binding energy within the target receptor was calculated to identify the most favorable binding pose.

### 2.4. Synthesis and Characterization of the FAP-Targeted NOTA-FAP-2286 Molecular Probe

DMF (15 mL/g) was initially added to pre-activate 2-Chlorotrityl Chloride Resin. A threefold excess of Fmoc-Cys(Trt)-OH, a tenfold excess of DIEA, and an appropriate amount of DMF were successively added to the resin and shaken for 30 min. Subsequently, methanol was added to cap the reaction, and the coupling of the first amino acid proceeded for an additional 30 min. After removing the solvent, Fmoc deprotection was achieved using 20% piperidine DMF (15 mL/g) for three cycles (1 × 5 min, 2 × 15 min). Following piperidine removal, ninhydrin testing confirmed the successful binding of the first amino acid to the resin. The resin was then washed sequentially with DMF (10 mL/g), methanol (10 mL/g), and DMF (10 mL/g), respectively. The remaining amino acids were coupled to the resin-bound peptides in a sequential manner from right to left, employing the standard protocol supplemented with the peptide coupling agent HBTU [22]. Next, peptides were cleaved from the resin for 180 min using a cleavage solution (10 mL/g) composed of 95% trifluoroacetic acid (TFA), 2% water, 2% ethanedithiol (EDT), and 1% triisopropylsilane (TIS). The cleavage solution was dried under nitrogen, and the crude peptides were precipitated with ether. Hex-Cys-Pro-Pro-Thr-Gln-Phe-Cys-OH was dissolved in sodium carbonate solution, followed by the addition of 1,3,5-tris(bromomethyl)benzene and mercaptoethylamine. Upon reaction completion, the crude product was purified by high-performance liquid chromatography (HPLC). The purified liquid was lyophilized to obtain Hex-[Cys(tMeBn(H-AET))-Pro-Pro-Thr-Gln-Phe-Cys]-OH. NOTA(tBu)_2_-FAP-2286 was cleaved using a lysis solution (10 mL/g) containing 95% TFA, 2% water, 2% EDT, and 1% TIS. The lysate was dried under nitrogen, and the crude peptide was precipitated with ether to yield NOTA-FAP-2286. The crude product was purified by HPLC, and the purified liquid was lyophilized into a white powder. The purity of NOTA-FAP-2286 was assessed by HPLC (Agilent 1260 infinity HPLC system, Agilent, Santa Clara, CA, USA) and electrospray mass spectrometry (EMS) (Waters Alliance Micromass ZQ 2000, Waters Corporation, Milford, MA, USA). The HPLC detection conditions were mobile phase A: acetonitrile with 0.1% TFA, mobile phase B: water with 0.1% TFA, gradient: 65–40–0% B over 0–20–20.1 min, and flow rate: 1 mL/min. The EMS conditions were capillary voltage: ±(2500–3500) V, desolvent flow rate: 800 L/h, desolvation temperature: 450 °C, cone voltage: 15–30 V, and run time: 1 min.

### 2.5. Preparation of the Al-Labeled NOTA-FAP-2286 Lyophilized Kit

To label Al^18^F-NOTA-FAP-2286, a lyophilized kit containing Al^3+^-chelated NOTA-FAP-2286, suitable for direct ^18^F-labeling, was initially prepared. A 1 mg aliquot of NOTA-FAP-2286, dissolved in 1 mL of 0.05 M NH_4_OAc buffer (pH 4.8), was combined with 198 µg of AlCl_3_·3H_2_O, dissolved in pure water. The mixture was reacted at 60 °C for 10 min. The product was purified using a pre-activated Sep-Pak C18 column and lyophilized into a vial containing 100 µg of NOTA-FAP-2286. The lyophilization process involved the following steps: pre-cooling at 4 °C for 30 min, freezing at −50 °C for 4 h, maintaining a vacuum of less than 50 Pa and a cold trap at −70 °C for 12 h during the first stage, and maintaining a vacuum of less than 50 Pa at 20 °C for 4 h during the second stage.

### 2.6. Synthesis and Characterization of Al^18^F-NOTA-FAP-2286

Approximately 200 µL of acetate buffer (pH 4.8) was used to dissolve [Al]-NOTA-FAP-2286. After loading ^18^F-ions onto the QMA column, it was thoroughly rinsed to remove potential metal ions. The QMA column was then rinsed with injectable saline, and 200 µL of the more concentrated ^18^F^-^ion eluate was collected and reserved. A 370 MBq aliquot of QMA-purified ^18^F^-^ions was added to the reaction mixture, and the high specific activity ^18^F-labeling reaction was conducted at 50 °C for 15 min. The entire synthesis process was performed in a dedicated synthetic hot cell equipped with a negative pressure system and radiation shielding, while the system’s pH was monitored before and after the reaction. The reaction mixture was separated on a Sep-Pak C18 column, and the free ^18^F- was removed by thorough elution. The target product, Al^18^F-NOTA-FAP-2286, was eluted with 50% ethanol and collected. It was subsequently diluted with saline, filtered through a sterile membrane, and prepared for use. To assess sample purity, radio-HPLC analysis was employed to specifically detect compound purity and RCP. The HPLC detection conditions were A: H_2_O-0.1% TFA, B: acetonitrile-0.1% TFA, gradient: 35–60–100–60% B (0–20.0–20.1–23.0 min), and flow rate: 1 mL/min.

### 2.7. Stability Test of Al^18^F-NOTA-FAP-2286

The purified Al^18^F-NOTA-FAP-2286 was filtered through a 0.22 µm sterile filter membrane and diluted with saline to achieve an ethanol concentration not exceeding 10% for subsequent injection. To evaluate in vitro stability, the injection was mixed with saline, 1% fetal bovine serum, or 0.01 M PBS, respectively, and incubated at 37 °C. Radio-HPLC was employed to monitor the RCP of Al^18^F-NOTA-FAP-2286 at 1, 2, and 3 h. In addition, injections containing the purified product were subjected to bacterial, endotoxin, and granulometry testing after 10 half-lives of storage.

### 2.8. Establishment of PRLT-Acquired Drug-Resistant Cells

RPMI-1640 complete medium containing ^177^Lu-PSMA-617 (50 kBq/mL) was used to culture 22RV-1 cells. After a 24 h incubation period in a 5% CO_2_ incubator at 37 °C, the medium containing ^177^Lu-PSMA-617 was discarded. The cells were washed twice with 1× PBS, and 10 mL of complete medium was added to the cell culture dish using a sterilized pipette. The dish was gently shaken, and the cells were returned to the 5% CO_2_ incubator at 37 °C for an additional 48 h incubation. This process was repeated three times to generate the PRLT-acquired drug-resistant cell line, designated as 22RV-1-R. A single PRLT treatment group is named L1. The multiple PRLT treatment-induced acquired drug-resistance group is named L3.

Cell lysates were collected after ^177^Lu-PSMA-617 treatment, and protein concentrations were measured by the BCA Protein Assay Kit (Shanghai Biyuntian Biotechnology Co., Ltd., Shanghai, China). The total proteins of 30 μg that were denatured were separated by SDS–polyacrylamide gel and transferred to the PVDF membrane by wet blotting. The membranes were blocked by 5% non-fat milk for 1 h and incubated with primary antibodies (PSMA and β-actin) overnight at 4 °C, and, subsequently, blots were incubated in peroxidase-conjugated secondary antibodies for 1 h at room temperature. Protein bands were detected by enhanced chemiluminescence (ECL) reagents (Thermo Scientific, MA, USA) according to the manufacturer’s instructions, the protein expression was quantified using Image J software (version 1.8.0.112, Rawak Software, Carlsbad, CA, USA).

For the cell uptake of ^177^Lu-PSMA-617, 1 × 10^5^ 22RV-1 or 22RV-1-R in the logarithmic growth phase were co-cultured with 0.37 MBq radiopharmaceuticals for 1 h. The medium was removed, and cells were washed with PBS three times. Cell uptake was quantified with a gamma counter.

For the cell internalization of ^177^Lu-PSMA-617, 1 × 10^5^ 22RV-1 or 22RV-1-R in the logarithmic growth phase were co-cultured with 0.37 MBq radiopharmaceuticals for 2 h. Remove the medium, rinse the cells twice with PBS, collect the supernatant and the rinse solution, and measure their radioactivity counts as the extracellular membrane radioactivity counts (A_outside_); add pre-cooled 0.5 mL of acetic acid buffer (pH 2.5) to each well in an ice bath twice, let it stand for 5 min each time, collect the supernatant into another new tube, and collect and measure their radioactivity counts as the cellular membrane radioactivity counts (A_membrane_); and in the Petri dish, add 0.5 mL of 1 mol/L sodium hydroxide solution to break the membrane for 30 min, repeat the procedure once, and collect and measure the radioactivity count of the supernatant as the inside cellular membrane radioactivity count (A_inside_). The radioactivity counts A_outside_, A_membrane_, and A_inside_ were measured with a γ-counter (Beijing PET Technology, China), respectively, and the rate of cell membrane internalization (%) = A_membrane_/(A_outside_ + A_membrane_ + A_inside_) × 100%.

### 2.9. Tumor Model

Healthy male athymic nude mice (BALB/c, nu/nu, 6–8 weeks old, 18–20 g) were purchased from Beijing Vital River Laboratory Animal Technology Co., Ltd. Animals were housed in individually ventilated cages under standard clean conditions with ad libitum access to food and water. The animals were maintained on a 12 h light/dark cycle at a temperature of approximately 21–22 °C and a humidity of 40–70%. The animal study protocol was approved by the Institutional Ethics Committee of Guizhou Medical University (No. 2305104, approval date: 16 November 2023).

After mixing 10^6^/100 µL of cell suspension (U87 cells or 22RV-1-R/NIH-3T3 co-cultured cells) with matrix gel at a 1:1 volume ratio, 0.2 mL of the cell suspension was aspirated into a syringe. Cells were injected subcutaneously into the right anterior thoracic region by inserting the needle through the skin and advancing it towards the forelimb. The needle was left in place for 1 min and then withdrawn, and the injection site was gently pressed. Once the animals regained consciousness, they were returned to their cages.

### 2.10. PET/CT Imaging of U87 Tumor-Bearing Mice

To verify the in vivo FAP targeting effect of Al^18^F-NOTA-FAP-2286, molecular imaging studies were conducted in U87 tumor-bearing mice. High-resolution images were acquired using a Siemens Inveon Micro PET/CT system. Mice were weighed and anesthetized with 3% isoflurane at an oxygen flow rate of 3 L/min. Following pre-anesthesia, 3.7 MBq of ^68^Ga-FAP-2286 (or Al^18^F-NOTA-FAP-2286) was intravenously injected via the tail vein. The mice were then placed in the scanning bed, which has a resolution of 1.5 mm, a single bed aperture of 5.7 cm, and an axial field of view (FOV) of 8.5 cm. For ^68^Ga-FAP-2286 PET, a single scan was performed at 45 min post-injection (p.i.), while multiple scans were acquired at 45 min, 90 min, 3 h, and 6 h p.i. for Al^18^F-NOTA-FAP-2286. For dynamic scans (2–90 min p.i.), mice were fixed in the prone position in the center of the FOV and maintained under continuous anesthesia with 1.5% isoflurane at an oxygen flow rate of 2 L/min. Image acquisition was performed using Inveon Acquisition Workplace (IAW) 1.5.0.28, which establishes a new acquisition process prior to data acquisition, including CT acquisition, CT reconstruction, PET acquisition, PET histogram generation, and PET reconstruction.

CT data acquisition, which followed the dynamic PET data acquisition, was performed at 80 kV tube voltage, 500 μA tube current, and 1100 ms exposure time for 10 min (5 min for each bed position).

Image reconstruction was accomplished using a three-dimensional ordered subset expectation maximization (OSEM3D) algorithm, followed by either maximization/maximum a posteriori (MAP) or fast MAP, as provided by IAW. Three-dimensional regions of interest (ROIs) were delineated over the lungs, heart, liver, kidney, intestine, muscle, and tumor, guided by CT images. Tracer uptake was subsequently quantified using the Inveon Research Workplace (IRW) 3.0. For each experimental set, three mice were utilized as parallel samples.

### 2.11. ^18^F-FDG PET/CT Imaging of U87 Tumor-Bearing Mice

For tumor localization and comparison with a clinically established imaging technique, ^18^F-FDG PET imaging was performed. Mice were fasted for 8 h and then administered 3.7 MBq of ^18^F-FDG intravenously via the tail vein. PET/CT imaging was conducted 45 min post-injection. The maximum standardized uptake value (SUV_max_) was automatically calculated by the IRW system using ROI analysis. Three mice were used as biological replicates for each experimental condition.

### 2.12. Al^18^F-NOTA-FAP-2286 PET/CT Imaging of 22RV-1-R/NIH-3T3 Co-Cultured Cell Tumor-Bearing Mice

For 22RV-1-R/NIH-3T3 co-cultured cell tumor-bearing mice, the Al^18^F-NOTA-FAP-2286 PET/CT scan employed the same injection dose and scanning procedure as detailed above for the U87 tumor-bearing mouse model, with the exception that only static imaging was performed. Upon completion of the scan, mice were euthanized via overdose anesthesia, and tumor tissues were harvested and preserved in 4% paraformaldehyde fixative for subsequent H&E staining histopathology and PSMA and FAP IHC assays (Yilianbo Technology Co., Ltd., Shanghai, China).

### 2.13. Statistical Analysis

Experimental data were processed using SPSS 21.0 and Origin 2024b software. Data were expressed as mean ± standard deviation (SD). Normality was assessed using the Shapiro–Wilk test. Student’s *t*-test was employed for comparisons between two groups of samples, while one-way ANOVA was used for comparisons among multiple groups. Statistical significance was determined at the *p* < 0.05 level. All experiments were performed in triplicate.

## 3. Results

### 3.1. Relationship Between FAP Expression and Prognosis

**Figure 1:**
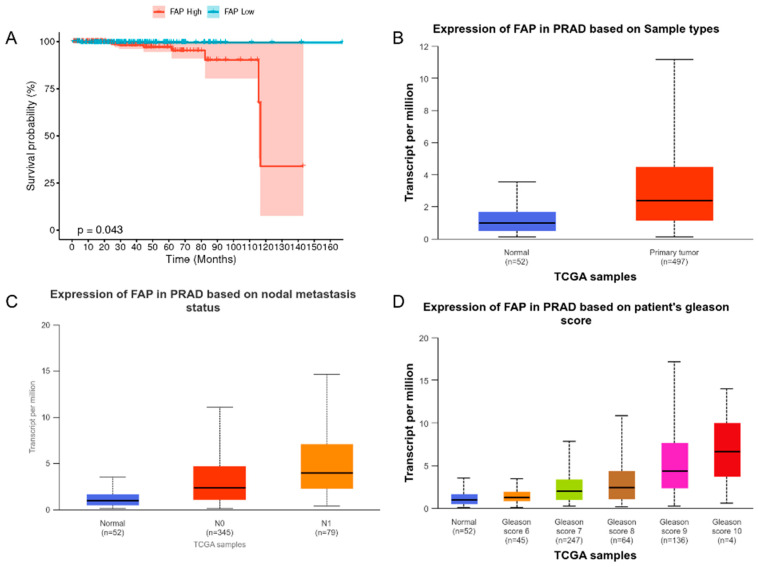
Relationship between FAP expression and prognosis. (**A**) Correlation between FAP expression and prognosis in PCa. (**B**) Comparison of FAP expression levels in normal prostate tissues and primary prostate cancer tissues. (**C**) Correlation analysis between FAP expression and lymph node metastatic status. (**D**) Distribution of FAP expression in prostate cancer tissues with different Gleason scores.

Figure 1A presents the results of a long-term follow-up study examining the relationship between FAP expression levels and the prognosis of PCa patients. The survival curve for the group with low FAP expression remained consistently near 100%, while the survival curve for the group with high FAP expression exhibited a gradual decline, particularly a significant drop around 110 months, ultimately reaching approximately 35%. A significant difference in survival was observed between the two groups (*p* < 0.05). These findings suggest that elevated FAP expression is associated with poorer long-term survival outcomes in PCa patients, providing compelling evidence for the potential utility of FAP as a prognostic marker for PCa.

In this study, the expression pattern of FAP in PCa progression and its potential clinical significance were analyzed in depth using the TCGA database. Figure 1B demonstrates that FAP exhibited significantly higher expression levels (*p* < 0.001) in primary PCa tissues (n = 497) compared to normal prostate tissues (n = 52), suggesting a potential involvement of FAP in PCa development.

Figure 1C further revealed a correlation between FAP expression and lymph node metastasis. Specifically, the expression level of FAP in patients with stage N1 (presence of lymph node metastasis) was significantly higher than that in patients with stage N0 (absence of lymph node metastasis) and in normal tissues (*p* < 0.01). This finding suggests that FAP may play a crucial role in the metastatic process of prostate cancer.

Figure 1D illustrates the correlation between FAP expression and Gleason score. The results reveal a progressive increase in FAP expression level with escalating Gleason score. Notably, FAP expression was significantly elevated in high-grade PCa (Gleason score ≥ 8) compared to the low-grade group (*p* < 0.001), strongly indicating a close association between FAP expression and PCa malignancy.

Collectively, these findings strongly support FAP as a potential biomarker for PCa progression and prognosis and provide a foundation for the in-depth exploration of the molecular mechanisms underlying FAP’s role in PCa development, metastasis, and malignant progression.

### 3.2. Correlation Analysis of FAP with Genes Associated with Radiotherapy Resistance

**Figure 2:**
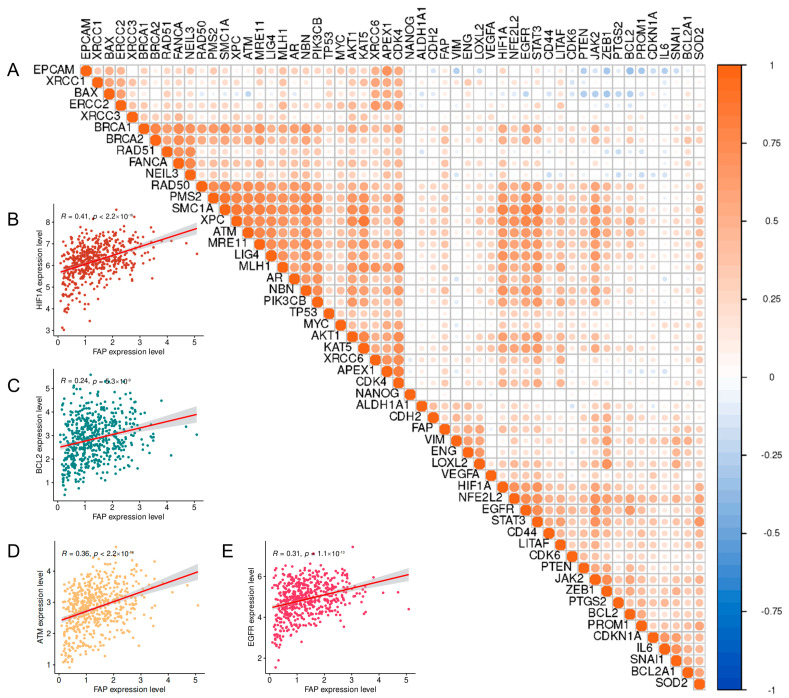
Correlation analysis of FAP with genes associated with radiotherapy resistance. (**A**) Heatmap analysis of the correlation between genes related to FAP and radiotherapy resistance. Correlation analysis of FAP expression and HIF1α (**B**), BCL2 (**C**), ATM (**D**), and EGFR (**E**) in PCa.

In this study, large-scale gene expression data analysis revealed a complex association network between FAP and multiple genes implicated in radiotherapy resistance, offering a novel perspective on FAP’s potential role in this phenomenon. Heatmap analysis (Figure 2A) demonstrated positive correlations between FAP and several key genes involved in DNA repair and cell cycle regulation. Notably, FAP exhibited strong positive correlations (r > 0.5, *p* < 0.001) with genes essential for homologous recombination repair, including *BRCA1*, *BRCA2*, *RAD51*, and *FANCA*, suggesting a potential role of FAP in enhancing DNA double-strand break repair and, consequently, contributing to radiotherapy resistance. In addition, FAP showed moderate positive correlations (0.3 < r < 0.5, *p* < 0.01) with cell cycle checkpoint-related genes, such as *ATM* and *TP53*, implying its involvement in regulating cell cycle progression and potentially influencing tumor cell sensitivity to radiotherapy.

Notably, FAP exhibited a positive correlation (r ≈ 0.4, *p* < 0.01) with genes involved in angiogenesis and tumor microenvironment remodeling, including *VEGFA*, *HIF1α*, and *EGFR*. This suggests that FAP may indirectly influence radiotherapy efficacy by modulating the tumor microenvironment, potentially promoting angiogenesis and the formation of a hypoxic microenvironment. Conversely, FAP displayed a weak to moderate negative correlation (−0.3 < r < 0, *p* < 0.05) with tumor suppressor genes, like *PTEN* and CDK inhibitors (e.g., *CDKN1A*), hinting at its potential role in driving tumor progression.

To further investigate the potential role of FAP in PCa radiotherapy resistance, we conducted detailed correlation analyses of FAP with four representative radiotherapy resistance-related genes (Figure 2B–E). These analyses not only corroborated the findings of the previous heatmap analysis (Figure 2A) but also provided more specific quantitative evidence supporting multiple mechanisms by which FAP contributes to PCa radiotherapy resistance. FAP exhibited the strongest positive correlation with *HIF1α* (R = 0.41, *p* < 2.2 × 10^−16^, Figure 2B). In PCa, a high expression of *HIF1α* is frequently associated with tumor hypoxia, radiotherapy resistance, and poor prognosis. This significant correlation strongly suggests that FAP may impact radiotherapy sensitivity by modulating the hypoxic response within the PCa microenvironment. FAP may contribute to aberrant tumor angiogenesis, leading to insufficient blood supply and the formation of hypoxic regions within the tumor, thus indirectly enhancing radiotherapy resistance.

FAP also exhibited a positive correlation with *BCL2* (R = 0.24, *p* = 6.3 × 10^−9^, Figure 2C), although the strength of this correlation was relatively weak. In PCa, high *BCL2* expression has been strongly associated with biochemical recurrence and disease progression following radiotherapy. This correlation suggests that FAP may potentiate the resistance of PCa cells to radiotherapy-induced cell death by upregulating the anti-apoptotic pathway.

The significant positive correlation between FAP and *ATM* (R = 0.36, *p* < 2.2 × 10^−16^, Figure 2D) further supports the potential role of FAP in DNA damage response. In PCa, the activation of *ATM* is associated with enhanced DNA repair and increased resistance to radiotherapy. The positive correlation between FAP and *ATM* suggests that FAP may contribute to radiotherapy resistance by enhancing the ability of PCa cells to repair radiation-induced DNA damage.

In addition, a significant positive correlation was observed between FAP and *EGFR* (R = 0.31, *p* = 1.1 × 10^−13^, Figure 2E). In PCa, the activation of the *EGFR* signaling pathway is closely linked to disease progression and resistance to radiotherapy. The positive correlation between FAP and *EGFR* suggests that FAP may influence the response of PCa cells to radiotherapy by modulating the *EGFR* signaling pathway, which may involve the promotion of cell proliferation, survival, and DNA repair through various mechanisms.

### 3.3. Induction and Validation of PRLT-Acquired Drug-Resistant Cells

**Figure 3:**
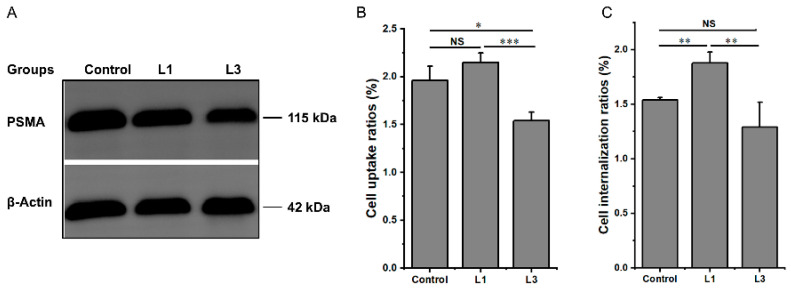
The changes in PSMA-related characteristics after treating 22RV-1 cells with ^177^Lu-PSMA-617 RLT. (**A**) Western blot analysis of PSMA expression level. (**B**) The ability of ^177^Lu-PSMA-617 uptake in different groups for 1 h. (**C**) The ability of ^177^Lu-PSMA-617 internalization in different groups for 2 h. NS: *p* > 0.05, * *p* < 0.05, ** *p* < 0.01, *** *p* < 0.001.

As can be seen in Figure 3A, the PSMA expression of 22RV-1-R cells obtained from multiple treatments and screening with ^177^Lu-PSMA-617 RLT did not fluctuate more dramatically and remained strongly positive for PSMA after three treatments with ^177^Lu-PSMA-617 RLT. The alteration in PSMA expression was also partially reflected in the dynamic change in the uptake capacity of 22RV-1-R cells for ^177^Lu-PSMA-617 dynamic change in the uptake capacity. Screening resulted in a reduction in the 1h uptake rate (1.54 ± 0.09% vs. 1.96 ± 0.15%, L3 vs. control, *p* < 0.05) (Figure 3B) and a reduction in the 2h internalization rate (1.29 ± 0.23% vs. 1.54 ± 0.02%, L3 vs. control, *p* > 0.05) (Figure 3C).

### 3.4. Molecular Docking Simulations of FAP Ligands

**Figure 4:**
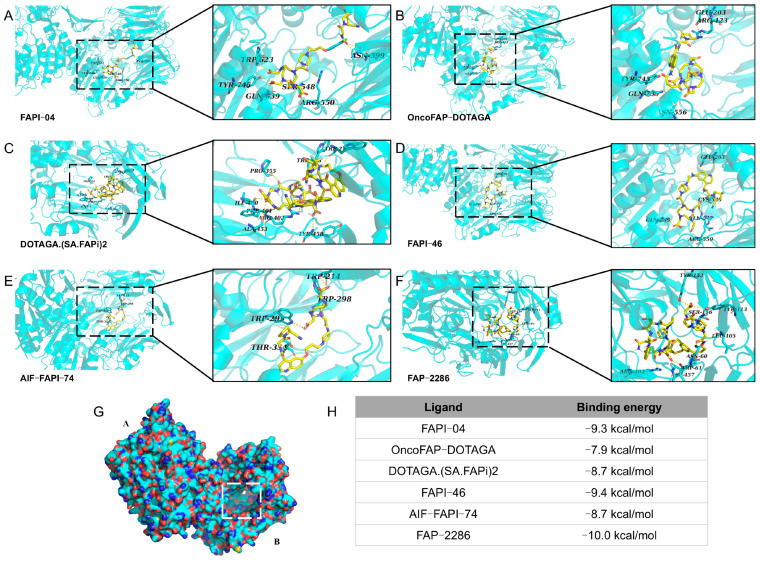
Molecular docking of FAP alpha and FAPI-04 (**A**), OncoFAP-DOTAGA (**B**), FAPI-46 (**C**), DOTAGA.SA.(FAPi)2 (**D**), FAPI-74 (**E**), and FAP-2286 (**F**). (**G**) Various FAP-targeting molecules and docking sites for FAP alpha. (**H**) Binding energies of various FAP-targeting molecules and FAP alpha.

Molecular docking simulations were performed using the PDB entry 1Z68, which represents the FAP alpha protein receptor. Autodock Vina 1.2.2 software was employed for docking various ligands with the receptor. FAPI-04 formed the most hydrogen bonds (seven) with A-chain residues Asn399 (2), Gln539, Ser548, Arg550, Trp623, and Tyr745 (Figure 4A). Other ligands displayed varying hydrogen bond interactions: OncoFAP-DOTAGA (six bonds with B-chain residues Arg123, Glu203, Gln539 (2), Asn556, and Tyr745, Figure 4B); FAPI-46 (five bonds with B-chain residues Glu203, Gln539, Cys545, Gln547, and Arg550, Figure 4C); DOTAGA.SA.(FAPi)2 (eleven bonds with A-chain residues Trp214, Trp298 (2), Pro355, Ile400, Phe401, Arg402 (3), Ala453, and Tyr458, Figure 4D); FAPI-74 (five bonds with B-chain residues Trp214, Trp295, Trp298, and Thr354 (2), Figure 4E); and FAP-2286 (eight bonds with B-chain residues Asn60, Trp61, Leu105, Tyr113, Tyr152, Ser156, Arg402, and Asp457, Figure 4F).

As depicted in Figure 4G,H, all docking sites were situated within the cavities of chain A or chain B. Among these, FAP-2286 exhibited the strongest binding affinity to FAP, with a calculated binding energy of −10.0 kcal/mol. Given the promising potential of FAP-2286 as an ^18^F-labeled imaging agent for detecting PRLT resistance and PSMA heterogeneity, it was selected for further experimental investigation.

### 3.5. Radiochemistry of Al^18^F-NOTA-FAPI-04

**Figure 5:**
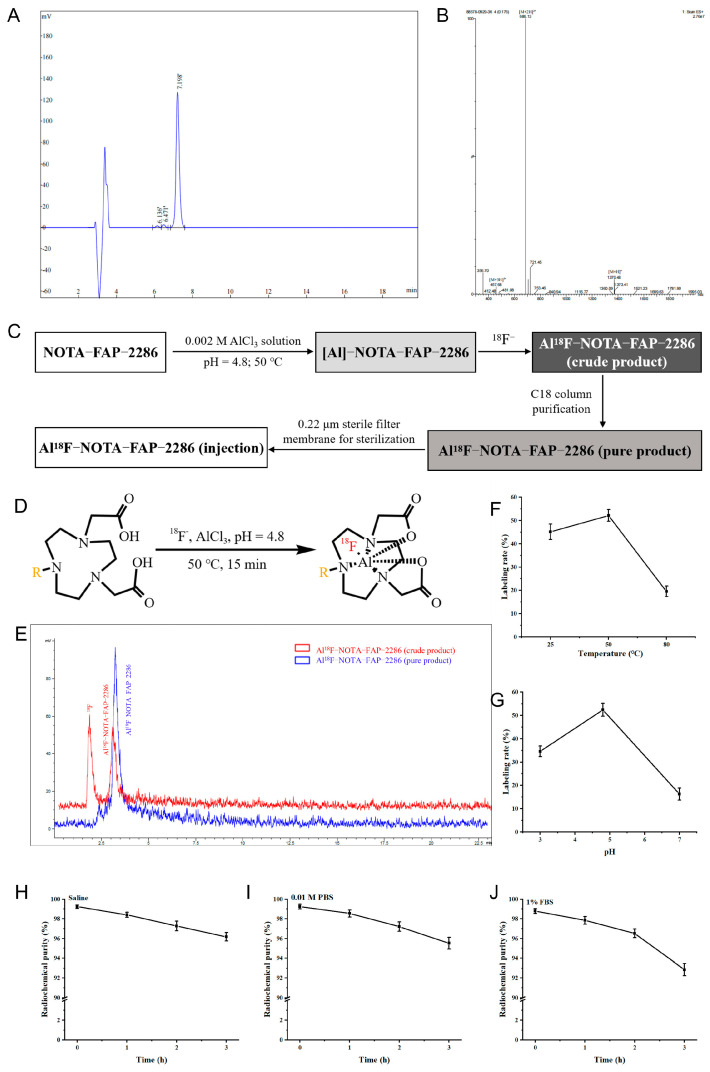
HPLC analysis of NOTA-FAP-2286 (**A**) and electrospray ionization mass spectrometry (**B**). (**C**) Synthesis, purification, and preparation process of injection of Al^18^F-NOTA-FAP-2286. (**D**) Coordination mode of NOTA and Al^18^F in the synthesis of Al^18^F-NOTA-FAP-2286 (R represents FAP-2286). (**E**) Radio–HPLC spectrum of Al^18^F-NOTA-FAP-2286 before and after purification. Effect of reaction temperature (**F**) and pH (**G**) on labeling efficiency of Al^18^F-NOTA-FAP-2286. The in vitro stability of Al^18^F-NOTA-FAP-2286 in saline (**H**), 0.01 M PBS (**I**), and 1% FBS (**J**) solution at 37 °C.

Based on prior molecular docking data, this study identified FAP-2286, a compound targeting tumor fibroblast components, as the molecule of interest. To facilitate imaging, the DOTA ring in FAP-2286 was substituted with a NOTA ring, resulting in NOTA-FAP-2286, which is more amenable to Al-^18^F labeling. As depicted in Figure 5A,B, NOTA-FAP-2286 exhibits a chemical purity of 96.81%, meeting the necessary criteria for use. The ESI-MS spectrum revealed peaks at [M + 2H]^2+^ = 686.13 and [M + H]^+^ = 1370.48, aligning with the theoretical molecular weight of 1369.68.

Figure 5C outlines the synthesis, purification, and preparation process for the injection of Al^18^F-NOTA-FAP-2286. Figure 5D depicts the coordination mode between NOTA and Al^18^F during the synthesis of Al^18^F-NOTA-FAP-2286. Under conditions of 50 °C and pH 4.8, the multiple batches labeled compound achieved a 48–55% labeling rate and an RCP exceeding 95% following polarity-based separation using a Sep-Pak C18 column. Radio-HPLC analysis revealed that the recovered Al^18^F-NOTA-FAP-2286 was a single component with a peak elution time of 3.26 min (Figure 5E). Notably, Al^18^F-NOTA-FAP-2286 was prepared in less than 30 min under mild conditions with high yield. As illustrated in Figure 5F, labeling rates at room temperature and 50 °C were 45.1 ± 3.3% and 52.1 ± 2.5% (pH = 4.8), respectively. In contrast, increasing the temperature to 80 °C (pH = 4.8) reduced the labeling rate to less than 20%. As shown in Figure 5G, the optimal labeling rate of 52.1 ± 2.5% was achieved at pH 4.8 with a fixed reaction temperature of 50 °C. Neutral conditions were unfavorable for Al^18^F labeling (16.3 ± 2.6%), potentially due to the formation of Al(OH)_3_ precipitate at the early stages of the reaction.

After a 3 h incubation with saline, 0.01 M PBS, or 1% FBS at 37 °C, the RCP of Al^18^F-NOTA-FAP-2286 remained > 90% (Figure 5H–J), indicating good in vitro stability. Retrospective quality testing of the injection confirmed adherence to established standards for bacterial content, endotoxin level, and particle size. No abnormalities were observed in mice post-injection, rendering it suitable for subsequent in vivo imaging experiments.

### 3.6. PET/CT Static and Dynamic Imaging of the U87 Tumor-Bearing Mice Model

**Figure 6:**
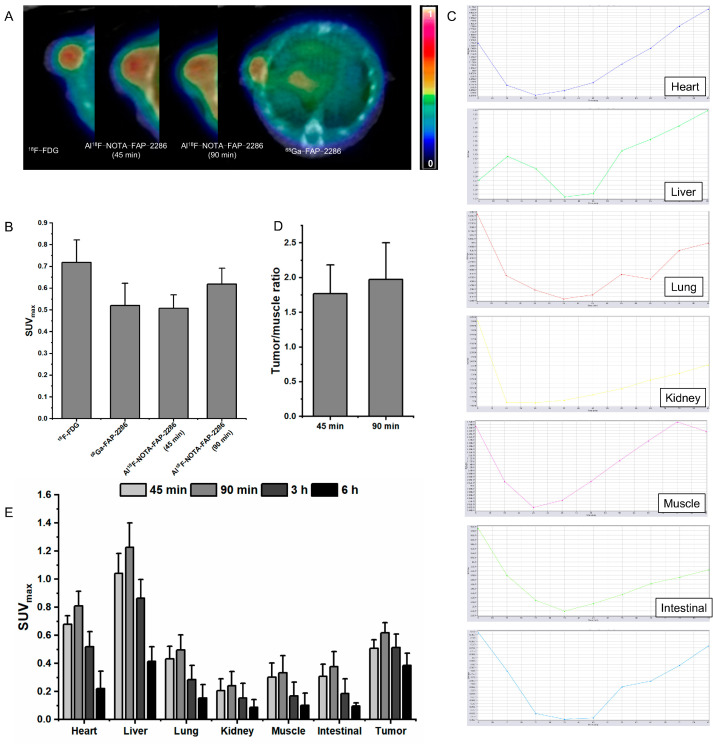
Representative PET/CT images (**A**) and the SUV_max_ of tumor (**B**) in ^18^F-FDG, Al^18^F-NOTA-FAP-2286 (45 min), Al^18^F-NOTA-FAP-2286 (90 min), and ^68^Ga-FAP-2286. (**C**) Al^18^F-NOTA-FAP-2286 dynamic uptake of different organs in U87 tumor-bearing mice. (**D**) Tumor/muscle ratio of U87 tumor mice to Al^18^F-NOTA-FAP-2286 at 45 min and 90 min. (**E**) Al^18^F-NOTA-FAP-2286 uptake of different organs at different time points.

Tumor cells exhibited elevated glucose metabolism, as evidenced by ^18^F-FDG PET/CT images acquired 45 min p.i., which revealed high radiotracer uptake at the tumor site with an SUV_max_ value of 0.718 ± 0.104. To verify FAP expression in a representative model, U87 xenografts were used to compare the tumor uptake of FAP-2286 and its analogs (Figure 6A,B). Leveraging the extended scanning window of F-18, acquired PET images at 90 min p.i. demonstrated increased tracer uptake (SUV_max_ = 0.508 ± 0.062 at 45 min p.i. vs. SUV_max_ = 0.619 ± 0.073 at 90 min p.i.). A 90 min dynamic PET imaging study was performed for each mouse, and the time–radioactivity uptake curve indicated that peak uptake of the Al^18^F-NOTA-FAP-2286 PET tracer by U87 mouse tumors occurred at 90 min (Figure 6C). Based on the target-to-normal tissue (tumor/muscle) ratio, the maximum value was observed at 90 min post-injection of Al^18^F-NOTA-FAP-2286 (Figure 6D). As depicted in Figure 6E, biodistribution demonstrated rapid clearance from non-target organs (liver SUV_max_ decreased from 1.228 ± 0.172 at 90 min to 0.416 ± 0.105 at 6 h) and stable tumor retention (SUV_max_ = 0.387 ± 0.087 at 6 h). In addition, most of the tracer was excreted from the kidneys at 6 h, demonstrating that renal clearance is the primary route. Low radioactivity in the muscle indicated more complete non-target clearance of Al^18^F-NOTA-FAP-2286, further contributing to improved target-to-normal ratios.

### 3.7. PET/CT Imaging of Al^18^F-NOTA-FAP-2286 in 22RV-1-R/NIH-3T3 Co-Cultured Cell Tumor-Bearing Mice

**Figure 7:**
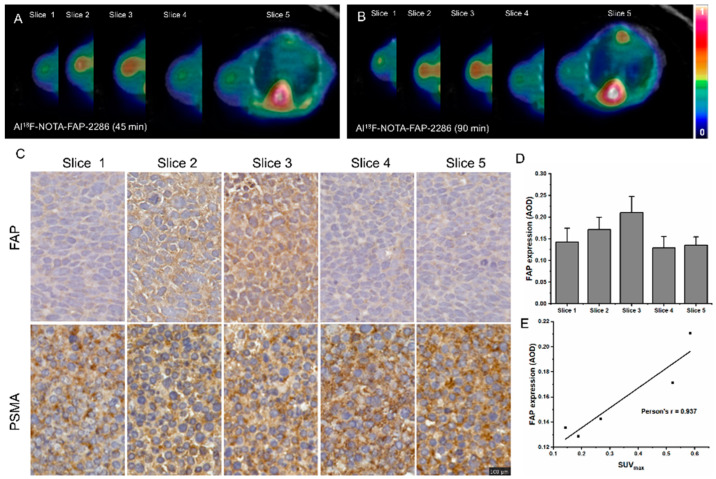
Representative Al^18^F-NOTA-FAP-2286 (45 min) (**A**) and Al^18^F-NOTA-FAP-2286 (90 min) (**B**) PET/CT images of different slices tumors in 22RV-1-R/NIH-3T3 co-cultured cell tumor-bearing mice. (**C**) Immunohistochemical PSMA and FAP staining of different slices in tumors in 22RV-1-R/NIH-3T3 co-cultured cell tumor-bearing mice (magnification × 200; scale bar: 50 μm). (**D**) Quantification of different slices in immunohistochemical sections of FAP. (**E**) Correlation between tissue uptake (SUV_max_) and FAP immunohistochemical expression in different slices of the tumors in 22RV-1-R/NIH-3T3 co-cultured cell tumor-bearing mice.

The initial applications of the ^18^F-labeled FAP-2286-derived imaging agent are illustrated in Figure 7, where Al^18^F-NOTA-FAP-2286 PET enabled mild imaging of the tumor region in 22RV-1-R/NIH-3T3 co-cultured cell tumor-bearing mice (SUV_max_ = 0.521 ± 0.096 at 45 min and SUV_max_ = 0.584 ± 0.103 at 90 min post-injection, Figure 7A,B). The Al^18^F-NOTA-FAP-2286 PET results indicate heterogeneous uptake at different tumor slices in 22RV-1-R/NIH-3T3 co-cultured cell tumor-bearing mice. These findings were corroborated by IHC staining for FAP (Figure 7C–E), which confirmed the correlation between FAP expression and imaging agent uptake in tumors. The quantitative relationship between Al^18^F-NOTA-FAP-2286 uptake, as determined by PET/CT imaging, and FAP IHC expression is summarized in Figure 7E. A strong positive correlation (r = 0.937, Figure 7E) was observed between the quantitative values of imaging agent uptake at different tumor slices and FAP IHC expression. This correlation further validates the specificity of Al^18^F-NOTA-FAP-2286 for FAP expression in acquired drug-resistant prostate cancer tumors.

## 4. Discussion

The highly heterogeneous nature of PCa and its varied biological responses to radiotherapy, coupled with the diversity of therapeutic options, pose significant challenges to clinical decision making. Accordingly, interdisciplinary collaboration is crucial. While diagnostic methods and validation studies for PCa are rapidly advancing, more systematic research is required to identify the most suitable imaging technique or combination of imaging modalities.

Imaging and intranuclear radiotherapy for FAP represent a promising strategy for the evaluation and treatment of malignant tumors [23]. While FAP imaging has shown potential complementary value to various imaging modalities, such as PSMA and SSA [5], extensive research-based data supporting its clinical utility remains limited. To expand the scope of nuclear medicine diagnosis for a diverse range of tumors, a series of FAP-targeted small molecule inhibitor-based FAPI precursors, including FAPI-02, FAPI-04, and FAPI-46, have been developed at the University Hospital of Heidelberg, Germany [16]. The NOTA-FAP-2286 peptide-targeted developer presented in this study incorporates a NOTA chelator while preserving the core FAP-2286-targeting structure, enabling efficient chelation of Al^3+^. This technological advancement is particularly significant given the widespread presence of tumor fibroblasts in various tumor tissues and their critical role in tumorigenesis and progression. Notably, FAP is overexpressed in fibroblasts of a wide range of malignant tumors, especially those with a strong connective tissue proliferative response, such as breast, colon, and pancreatic cancers. This finding underscores the broad oncological potential of FAP-2286 as a drug delivery system. Overall, NOTA-FAP-2286, with its optimized Al^3+^ chelating ability and FAP-targeting properties, coupled with the widespread overexpression of FAP in tumors, provides a strong foundation for broad-spectrum oncological efficacy.

In this work, Al^18^F-NOTA-FAP-2286 was successfully synthesized, and preliminary analysis identified the primary factors influencing its labeling. The degree of metal ion separation from the QMA column was the primary factor affecting the labeling rate, while the degree of ^18^F removal from the labeling system via the C18 column was the primary factor preventing bone uptake. Chelation-based ^18^F labeling offers a simpler approach compared to conventional nucleophilic or electrophilic reaction steps, facilitating the potential scale-up of the labeling system. Al^18^F-NOTA-FAP-2286 exhibits a longer half-life than the commonly used Ga-68, making it suitable for scenarios with stringent imaging time requirements. This improvement not only enhances imaging flexibility but also expands the potential for detecting a wider range of tumor types in clinical practice.

In preclinical studies, ^68^Ga-FAPI PET/CT has demonstrated tumor specificity in 28 tumor types [24,25]. In addition, both the previous and current studies consistently showed that FAPI-based diagnostic agents exhibit lower background uptake in the brain, liver, and oropharyngeal mucosa compared to conventional FDG PET imaging [26]. This property enables Al^18^F-NOTA-FAP-2286 to achieve a higher lesion detection rate when targeting tumors in these regions. More importantly, the high specificity and rapid off-target organ clearance of this targeting molecule provide a theoretical foundation for its use in tumor therapy in combination with therapeutic nuclides such as ^90^Y, ^177^Lu, or ^225^Ac. This combination strategy can maximize the protection of non-target organs from ionizing radiation, facilitate precise radiation dose planning, and reduce the required drug radioactivity doses for treatment, thereby improving therapy safety and efficacy [27].

The experimental animal model used in this study exhibited dynamic changes in PSMA and FAP expression as the tumor progressed, underscoring the importance of timely tissue sampling for IHC analysis in clinical practice. This finding suggests that Al^18^F-NOTA-FAP-2286 can provide additional diagnostic information, aiding clinicians in gaining a more comprehensive understanding of a patient’s condition, particularly when PSMA expression assessments are inadequate or uncertain. The clinical application of Al^18^F-NOTA-FAP-2286 has significantly improved the diagnostic accuracy and staging precision of PCa. As a PSMA expression-independent visualizer, Al^18^F-NOTA-FAP-2286 offers a unique complementary diagnostic value. This property makes it a valuable molecular imaging tool for diagnosing PCa progression, especially when assessments following PRLT are insufficient. However, it is crucial to emphasize that Al^18^F-NOTA-FAP-2286 is not a complete substitute for ^68^Ga-labeled PSMA visualizers. In PCa cases with high PSMA expression, PSMA-like peptide visualizers retain higher specificity and detection rates. Therefore, in clinical practice, imaging agents should be rationally selected based on specific patient conditions and diagnostic needs. A combined approach using Al^18^F-NOTA-FAP-2286 and PSMA imaging agents may enhance diagnostic accuracy and comprehensiveness.

## 5. Conclusions

In this study, Al^18^F-NOTA-FAP-2286 was synthesized through a facile pre-chelation of Al^3+^, resulting in enhanced marker stability to meet clinical translation requirements. Preliminary clinical trials confirmed that the extended half-life enables a longer imaging observation window, providing the potential for increased diagnostic information. The imaging agent was prepared under mild conditions, demonstrating both high yield and good stability in in vitro experiments. To further optimize the synthesis conditions of Al^18^F-NOTA-FAP-2286, the effects of temperature and pH on its labeling rate were meticulously investigated. A high labeling rate was achieved for this developer at 50 °C and pH 4.8. Preliminary imaging experiments confirmed the potential of Al^18^F-NOTA-FFAP-2286 for subsequent PSMA heterogeneity imaging.

## Data Availability

All data generated or analyzed during this study are included in this published article.

## Abbreviations

The following abbreviations are used in this manuscript:

PCa	Prostate cancer
PSMA	Prostate-specific membrane antigen
PET	Positron emission tomography
^99m^Tc-MDP	Technetium-99m-methyl-diphosphonate
PRLT	PSMA radioligand therapy
IHC	Immunohistochemistry
PSA	Prostate-specific antigen
FAP	Fibroblast activation protein-α
FAPI	FAP inhibitor
RCP	Radiochemical purity
PRAD	Prostate adenocarcinoma
RNA-Seq	RNA sequencing
TCGA	The Cancer Genome Atlas program
TPM	Transcript per million
FPKM	Fragment per kilobase of transcript per million
HCO	Hierarchical clustering order
HIF1α	Hypoxia-inducible factor 1α
BCL2	B-cell lymphoma 2
ATM	ATM serine/threonine kinase
EGFR	Epidermal growth factor receptor
PDB	Protein Data Bank
LGA	Lamarckian genetic algorithm
HPLC	High-performance liquid chromatography
EMS	Electrospray mass spectrometry
TFA	Trifluoroacetic acid
EDT	Ethanedithiol
TIS	Triisopropylsilane
FOV	Field of view
p.i.	Post-injection
IAW	Inveon acquisition workplace
OSEM3D	Three-dimensional ordered subset expectation maximum
MAP	Maximization/maximum a posteriori
ROIs	Regions of interest
IRW	Inveon research workplace
SUV_max_	Maximum standardized uptake value
SD	Standard deviation
